# An Application of the Runs Test to Test for Randomness of Observations Obtained from a Clinical Survey in an Ordered Population

**DOI:** 10.21315/mjms2018.25.4.15

**Published:** 2018-08-30

**Authors:** Mohamad Adam Bujang, Fatin Ellisya Sapri

**Affiliations:** Clinical Research Centre, Sarawak General Hospital, Ministry of Health, Malaysia

**Keywords:** random, runs, sample, sampling, survey

## Abstract

Runs test is a statistical procedure which determines whether a sequence of data within a given distribution have been derived with a random process or not. It may be applied to test the randomness of data in a survey that collect data from an ordered population. This article illustrates on method to perform a runs test and explains the rationale for performing it by providing some examples of how this test can be applied. The aim of this article was to describe on ways to use the runs test in a clinical survey from an ordered population to determine the degree of randomness in the sequence of subjects who are recruited within a sample obtained from the whole population. Clinical survey that involves an ordered population usually collects data from subjects who have been recruited by a consecutive sampling method. Therefore, this study recommends that the degree of randomness in the sequence of selected variable(s) obtained from consecutive sampling is necessary to be tested from a pilot study to ensure random data collection in the study.

## Introduction

The terms ‘random’ and ‘non-random’ are commonly used to describe whether data have been obtained using a random or a non-random sampling procedure (1). For a clinical survey, a random sample of data will be collected if the probability sampling method has been adopted (2). On the other hand, although it is still possible for non-probability sampling to generate a random data, there is, however, no assurance that the sequence of data generated will be random because there is no mechanism to ensure random selection.

The present article emphasised sample selection from clinical survey in an ordered population. An ordered population is based on a ‘first come, first served’ manner. This type of population is usually occurring from a queue system such as when the patients are queuing while waiting to be treated by physicians or the cars are queuing for making an order at fast food restaurants. Among those sampling techniques under non-probability sampling, consecutive sampling is the commonest and easiest technique for an ordered population. Since consecutive sampling is one of the non-probability sampling techniques, it is necessary to check whether or not a random data has been collected from the process of recruitment. This is because statistical inferences drawn from a population using inferential statistics can only be valid if sample data have been collected without bias (3).

## The Concept and Application of Runs Test

A run is a sequence of events of a certain type preceded and followed by occurrences of the alternate type or by no events at all. A sample with too many or too few runs suggests that the sample may not be random. The runs test is a statistical test to determine whether random selection has been made in the process of sample selection from an ordered population. The runs test is a type of non-parametric test and hence, there is no need for the assumption of a normal distribution to hold true. It has been stated that for one sample test, the variable must be dichotomous while for two-sample test, the two samples should be mutually independent (4). This article aimed to describe the procedures in performing the runs test, and then illustrate how this test can be applied by using some real-life examples. The application of runs test in testing the randomness in the selection can become an evidence to show that there is no bias in the selection process.

## Hypotheses

There are several hypotheses can be tested using the runs test. The researcher has to select the correct hypothesis statement depending on which hypothesis the researcher wishes to test. The various hypothesis statements are as follows:

1. Two-sided
  H_0_: The occurrence of pattern for the two types of the observations is determined by a random process.
  H_1_: The occurrence of pattern for the two types of the observation is not determined by a random process.
2. One-sided
  H_0_: The occurrence of pattern for the two types of the observations is determined by a random process.
  H_1_: The occurrence of pattern for the two types of the observation is not determined by a random process because there are too few runs.
3. One-sided
  H_0_: The occurrence of pattern for the two types of the observations is determined by a random process.
  H_1_: The occurrence of pattern for the two types of the observation is not determined by a random process because there are too many runs.

Say a researcher observes a sequence of 20 patients attending at clinic A. The researcher would now like to determine whether the sequence is following a random sequence based on gender (M= Male and F= female).

F F M M F M F F F F M F F M M M M M F F

First of all, the researcher has set the hypothesis with two-sided test. The hypothesis with two-sided test can now be re-defined to make it more precisely reflecting the specific scenario for the hypothesis testing, as shown below:

H_0_: The pattern of sequence in terms of gender attended at clinic A is arranged in random manner.
H_1_: The pattern of sequence in terms of gender attended at clinic A is arranged in non-random manner.

Next is to count the number of observations for female, male and the number of runs for this series:

A run is defined as a series of consecutive observations’ values. In this example, the researcher shall count number of consecutive for both M and F. The calculation for the runs is as follow;

$$\begin{array}{l} \text{Runs}:\underset{1}{\underset{︸}{\text{F F}}}\;\underset{2}{\underset{︸}{\text{M M}}}\;\underset{3}{\underset{︸}{\text{F}}}\;\underset{4}{\underset{︸}{\text{M}}}\;\underset{5}{\underset{︸}{\text{F F F F}}}\;\underset{6}{\underset{︸}{\text{M}}}\;\underset{7}{\underset{︸}{\text{F F}}}\\ \text{Runs}:\underset{8}{\underset{︸}{\text{M M M M M}}}\;\underset{9}{\underset{︸}{\text{F F}}} \end{array}$$

Therefore, number of female (*n_1_*) = 11, number of male (*n_2_*) = 9 and runs (*r*) = 9.

## Test Statistics

Next, is to calculate the test statistic. Test statistic is a standardised value that is calculated from sample data for a hypothesis test which measures the agreement in between the sample data. The calculated value of the test statistic is used to compare the data obtained from the experimental conditions with the data expected to be obtained if the null hypothesis is valid. Therefore, the test statistic is used to determine whether or not the null hypothesis should be rejected. The methods of calculation for both test statistics and critical values are different between a small sample and a large sample.

Generally, test statistics for small sample will be based on the number of runs, while approximation technique will be applied for large sample. Besides, critical values for small sample will be obtained from a runs test for randomness table while a formula will be used to generate critical values for large sample. The sample sizes are considered as small when both *n_1_* and *n_2_* are 20 or less (1, 5–6). In other words, it is recommended that, for sample size more than 20 to apply approximation method.

Therefore, the following example will be considered to be a small sample because the observations are 20. Thus, the test statistics for a small sample is considered equivalent to the number of runs. Referring to previous example, there are 9 runs in event 1 and event 2, therefore test statistics is *r* = 9.

## Critical Value for Small Samples

The critical value can be obtained from table by Swed and Eisenhart (1). Hence, from the table, with *n_1_* = 11 and *n_2_* = 9, the upper critical value (Uc) obtained was six and the lower critical value (Lc) obtained was 16.

## Decision Rule

The following is the decision rule for a small sample based on the different types of hypothesis statements:

1. Two-sided
  Reject H_0_:
  *r* ≤ Lc or *r* ≥ Uc
2. One-sided (H_1_ has too few runs)
  Reject H_0_:
  *r* ≤ Lc
3. One-sided (H_1_ has too many runs)
  Reject H_0_:
  *r* ≥ Uc

From this example, since 6 ≤ *r* = 9 ≤ 16, hence, the decision is to accept H_0_. Therefore, we can conclude that there is enough evidence to claim that the sequence in the gender of patients attending clinic A had been selected in a random manner.

## Approximation Technique for Large Samples

The next discussion is to describe on how to perform the runs test for large samples of numerical (or continuous) data. Let’s take an example of HbA1c level obtained from patients with diabetes mellitus in clinic B. The data-set are as follow:

7.7, 10.0, 8.8, 12.1, 7.4, 6.1, 6.1, 6.1, 8.0, 8.8,

6.1, 10.9, 5.0, 12.6, 11.7, 11.5, 6.4, 8.9, 7.2, 7.2,

6.5, 10.3, 7.9, 7.8, 14.0, 6.8, 7.7, 7.8, 6.7, 7.7

For numerical data, researcher will need to first categorise it into two groups based on certain cut-off points. The cut-off points can be obtained from mean, median, mode or by custom (as determined by the researcher). Cases with values less than the cut-off point are categorised in one group while cases with values more than the cut-off point are categorised into another group. In this example, the researcher will use the custom cut-off point of 7, HbA1c < 7 as group 1 indicating good control and HbA1c ≥ 7 as group 2 which indicates poor control. The data were grouped as follows:

2 2 2 2 2 1 1 1 2 2 1 2 1 2 2 2 1 2 2 2 1 2 2 2 2 1

2 2 1 2

Next, the researcher need to set hypotheses for this analysis. Let’s say the null hypothesis is the pattern of sequence in terms of HbA1c level (poor or good control) observed at clinic B is arranged in random manner. Then, the next step is to calculate the test statistic. For a large sample, the test statistic can be calculated by using an approximation of the normal distribution via the following formula (7):

(1) $$z=\frac{r-\mu_{r}}{\sigma^{r}}$$

where:

*r* is the number of runs;
*μ_r_* is the expected number of runs; and
*σ_r_* is the standard deviation of the number of runs.

The values of *μ_r_* and *σ_r_* are computed as follows:

(2) $$\mu_{r}=\frac{2n_{1}n_{2}}{n_{1}+n_{2}}+1$$

(3) $$\sigma_{r}=\sqrt{\frac{\left(2n_{1}n_{2}\right)\left(2n_{1}n_{2}-n_{1}-n_{2}\right)}{{\left(n_{1}+n_{2}\right)}^{2}\left(n_{1}+n_{2}-1\right)}}$$

Let’s calculate for mean and standard deviation of the runs:

$$\begin{array}{l} \mu_{r}=\frac{2(9)(21)}{9+21}+1\\ =13.6 \end{array}$$

$$\begin{array}{l} \sigma_{r}=\frac{\left[2(9)(21)][2(9)(21)-9-21\right]}{{\left(9+21\right)}^{2}9+21-(1)}\\ =\sqrt{5.04}\\ =2.245 \end{array}$$

Test statistics:

$$\begin{array}{l} z=\frac{15-13.6}{2.245}\\ =0.624 \end{array}$$

After that, the researcher need to find the critical value for large sample. So, the calculation for the critical value is as follow:

$$\begin{array}{l} \text{Critical value }(\text{two tail})=z_{1}-\frac{\alpha }{2}\\ =z_{1}-\frac{0.05}{2}\\ =z_{0.975}\\ =1.96 \end{array}$$

At the 5% significance level, a test statistic with an absolute value greater than 1.96 indicates non-randomness (reject H_0_). For this example, since the test statistics < 1.96, hence the analysis accept H_0_. Therefore, we can conclude that there is enough evidence to claim that the sequence of subjects taken for HbA1c was in a random manner.

It is recommended to use z-score while handling a large sample or when the number of observations is more than 20. This is because the distribution of the observed number of runs would approximately follow the normal distribution that has a mean of zero and variance of one (5). If researchers want to do one-tailed runs test (for the purpose of detecting many runs), then researchers need to compare the z-score with upper tail critical value. Although the steps to obtain the critical values for lower tail and upper tail (for a one-tailed runs test) are the same as those for a two-tailed runs test, however different formula should be applied for one-tailed runs test, as shown below:

$$\begin{array}{l} \text{Critical value }(\text{upper tail})=z_{1}-\alpha \\ =z_{1}-0.05\\ =z_{0.95}\\ =1.645 \end{array}$$

For lower-tail critical value, just simply add negative sign on the critical value.

## Using the Runs Test to Test for Randomness of Observations Obtained from a Clinical Survey of an Ordered Population

There is a risk of selection bias when collecting a sample using consecutive sampling for an ordered population. Hence, it is strongly recommended to provide evidence with a runs test to demonstrate that the sequence of data is random. The runs test is useful for sampling situations in which there is an ordered sampling frame, such as in the queue system or where there is an interval between two consecutive observations (such as the presence of a specified period of waiting time between them). This is where consecutive sampling method which is based on the principle of ‘first-come-first-served’ commonly adopted.

In consecutive sampling, the sample collection is performed by screening each subject consecutively and including every subject who meets all inclusion criteria until the required sample size has been achieved (8). Unlike convenience sampling where the sample selection is selected based on the sample that is accessible by the researchers irrespective of whether it is a consecutive selection or not (2). Both are non-probability sampling methods and hence, there will always be a possibility for selection bias to occur (2).

Example, a researcher is conducting a research study to measure quality of life among patients with end stage renal disease (ESRD) who are attending routine follow-up in a clinic. In this scenario, the population is arranged in order and therefore, one of the easiest nonprobability sampling techniques in recruiting the patients is by using the consecutive sampling method. Assume that the quality of life was hypothesised to be decreasing as the respondents get older (9), so if the researcher recruits the sample by using consecutive sampling and the sample yields majority of younger respondents in the sample, so the measurement for quality of life may not be representative to the entire patient population. Therefore, this scenario explains on the situations where it is necessary to test the randomness in the selection process.

However, it is not necessary to test whether there is random sequence for all variables because only those relevant variables must be randomly sampled. For the measurement of quality of life among ESRD patients, a good starting point shall be to evaluate whether (or not) the age of the sample of patients who have been recruited for a clinical survey are in a random sequence. In addition, it will not be necessary to determine that all the samples collected are random. This is because the most important requirement is to ensure that the sample collection process is random.

Hence, this article proposes that the runs test has to be performed during a pilot study to ensure whether there is a random order in the age of those patients coming to the clinic. It is not recommended to perform the runs test in a full-scale research because the *P*-value will then become too small if the sample size is large, which will make it unnecessary to conduct the test (since the small *P*-value is resulting from a large sample size, and is not due to the presence of a real statistical significance). So, this study recommends to collect only a small sample (20 to 30 subjects) to determine whether there is a random sequence of the sample collected for a clinical survey or not.

Before performing the runs test, it is first necessary to get a clear understanding of the particular aspect of the target population. For example, says the researcher has already known that the prevalence of older people with ESRD is much higher than that of younger people, and so the order for arrangement of patients’ age may not be random as it is more likely for the first 10 or 20 patients collected within one sample to be all elderly. In this case, it is expected that the order of patients’ age to be likely not random. In this situation, it is necessary to collect a larger sample for pilot study such as 30 to 50 subjects per sample.

To conduct a pilot study using a large sample containing between 30 and 50 subjects can be difficult for certain surveys, hence, there is an alternative by using patients’ age from the past data. This is feasible if the clinic has kept a database which reports the history of patients’ notification by the clinic in a ‘first-come-first-served’ manner. By retrieving past data from a clinic, it is feasible for us to determine the random order of patients’ data, provided that there is no difference in the practice of recording patients’ data between the past and the present. By doing so, researchers can save both time and cost of the study.

If it has been subsequently found that the selection process using the consecutive sampling method is not random, then further investigation must be conducted to determine whether or not the selection bias can be due to the presence of a consecutive pattern which exists in the sequence of subjects recruited for a sample. This consecutive pattern may exist because of prior arrangements made at the clinics such as the treatment of severe cases (who are also usually older patients) during the morning and the treatment of less severe cases (who are also usually younger patients) in the afternoon. If this is occurring, it is now necessary to take corrective action to overcome the lack of random sequence of sample data collection by revising the study design including the selection of sampling technique.

Another proposed solution to address the above problem is to recruit a larger sample for the clinical survey. Previous studies have found that a survey which aims to recruit a minimum sample size of 300 subjects will be very likely to provide the estimates derived from the samples which closely mimic those obtained from the real population parameters (10–11). In other words, it nullifies the presence of any consecutive patterns within the sample. Moreover, a large sample size obtained will make it more likely for the estimates derived from the sample to be closer approximation to the real population parameters, which is in line with the concept of central limit theorem (12–13).

As for conclusion, this study recommends that the randomness of observations or data derived from consecutive sampling need to be assessed from a pilot study by using runs test before collecting data in the fieldwork phase of the study. This is to ensure no bias is implemented in the process of subject recruitment. Besides, runs test can also be applied to determine whether or not there is a consecutive pattern in the sample recruitment before implementing systematic sampling. Corrective actions need to be made if no-nrandom pattern is observed such as re-designing the sampling technique or revising the inclusion and exclusion criteria of sample recruitment.
